# Data resource profile: Exercise habits, step counts, and sedentary behavior from the National Health and Nutrition Survey in Japan

**DOI:** 10.1016/j.dib.2024.110103

**Published:** 2024-01-24

**Authors:** Takashi Nakagata, Rei Ono

**Affiliations:** a Department of Physical Activity Research, National Institutes of Biomedical Innovation, Health and Nutrition, Osaka, Japan; b Laboratory of Gut Microbiome for Health, Microbial Research Center for Health and Medicine, National Institutes of Biomedical Innovation, Health and Nutrition, Osaka, Japan; c Department of Public Health, Kobe University Graduate School of Health Science, Hyogo, Japan

**Keywords:** Health Japan 21, Population-level trends, Physical activity, Surveillance

## Abstract

The National Health and Nutrition Survey consistently assesses the prevalence of exercise habits, step counts, and sedentary behaviors in a strategically selected random sample of the Japanese population. The aim of this study was to provide descriptive epidemiological data on the average frequency of exercise habits, daily step counts, and sedentary behavior among Japanese adults from 2003 to 2019 using the National Health and Nutrition Survey database in Japan. Data were obtained from electronically available aggregate reports on the official survey website. To prepare for the third term of Health Japan 21, scheduled to start in 2024, we summarized population-level trends in exercise habits, step counts, and sedentary behavior among Japanese adults. The results could improve our understanding of trends in physical activity with respect to age and gender, providing a basis for public health monitoring and policy-making.

Specifications Table

**Table:** 

Subject	Public Health and Health Policy
Specific subject area	Exercise habits, step counts and sedentary behavior data in Japan
Data format	Raw, Analyzed, Filtered
Type of data	Tables, Figures
Data collection	Downloaded from e-Stat Portal Site of Official Statistics of Japan
Data source location	National Health and Nutrition Survey in Japan has been conducted by Ministry of Health, Labour and Welfare. 1-2-2 Kasumigaseki Chiyoda-ku Tokyo, 100-8916 Japan
Data accessibility	Repository: Mendeley Data Direct link to the data: https://data.mendeley.com/datasets/66tngkf6zf/1 Persistent identifier of the data: 10.17632/66tngkf6zf.1

## 1 Value of the Data

• These data show the long-term trends of the prevalence of exercise habits, step counts using a pedometer from 2003 to 2019, and sedentary behavior as physical activity parameters.
• Researchers, clinicians, and policymakers should be aware that, since 2003, average step counts have been decreasing, and there is variability in step counts among prefectures.
• These results provide an important perspective when considering the trends and development of exercise habits and step counts, which are set as the goals of Healthy Japan 21.

## 2 Data Description

In Japan, the National Health and Nutrition Survey (NHNS), formerly known as the National Nutrition Survey (NNS), has been conducted annually since 1945 to assess the physical condition, nutritional intake, and lifestyle habits of the population [1]. Surveys for 2020 and 2021 were suspended owing to the impact of the COVID-19 pandemic; the sampling design and survey items were changed to focus on public health issues during that period [2]. However, this survey continuously evaluated the prevalence of exercise habits, step counts using a pedometer, and sedentary behavior as physical activity parameters. Previous studies have independently examined population-level trends in exercise habits and step counts among Japanese adults. For example, Inoue et al. systematically examined step counts from 1995 to 2007 [3], Nishi et al. investigated step counts and exercise habits from 2003 to 2010 [4], and Takamiya and Inoue examined step counts from 1995 to 2016 [5]. However, reports on sedentary behavior and comprehensive studies of exercise habits, step counts, and sedentary behavior as well as specific regional differences are lacking.

An analysis of trends in exercise habits, daily step counts, and the distribution of sedentary behavior could provide important data for policymakers and relevant stakeholders to understand and forecast the health status of the population and facilitate policy-making to improve population health. Therefore, the aim of this study was to provide descriptive data on ambulatory physical activity in a sample of Japanese adults, including a summary of survey items from the NHNS. This paper summarizes the history of the NHNS from its origins to the present and provides descriptive data on ambulatory physical activity in Japanese adults.

## 3 Experimental Design, Materials and Methods

**Fig. 1:**
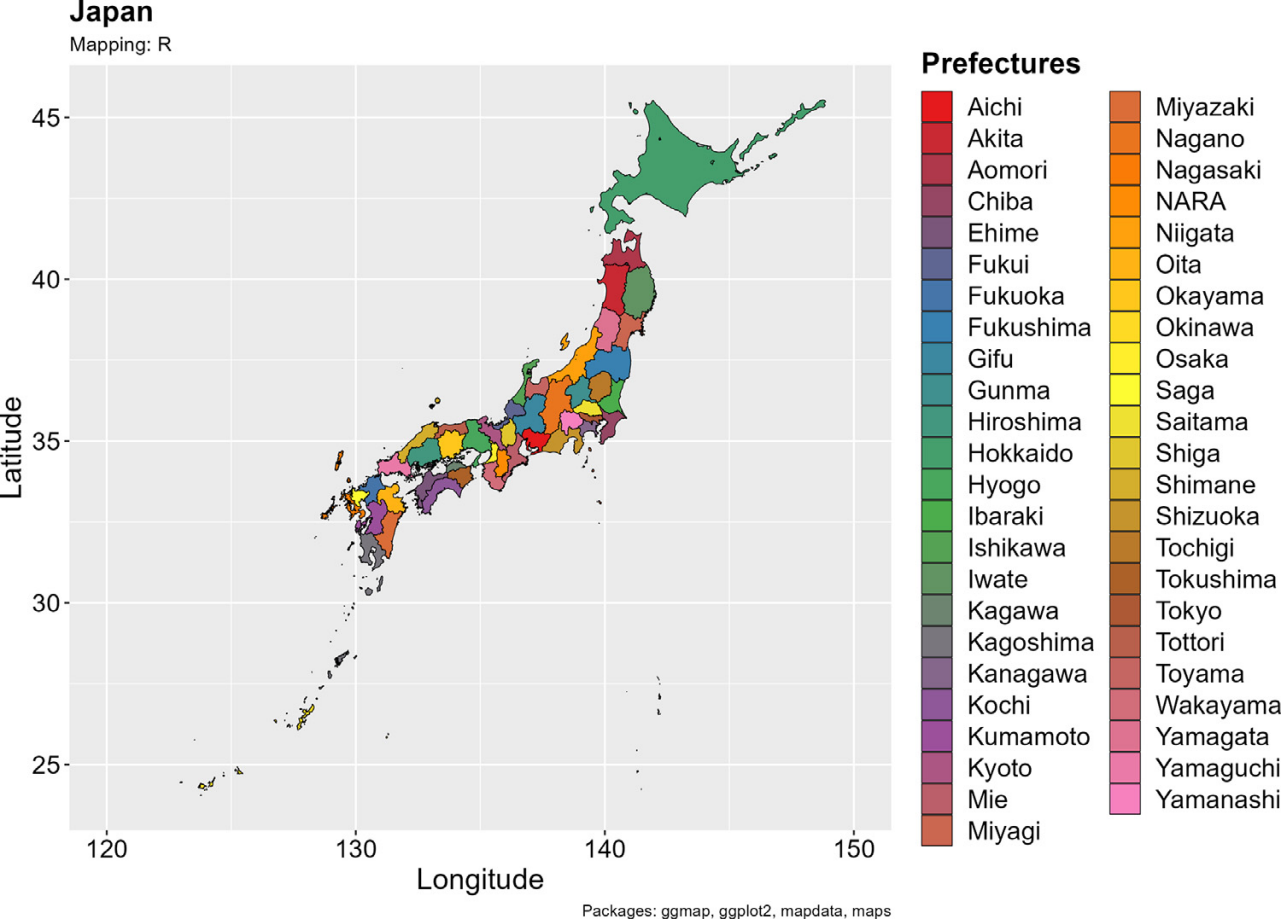
Mapping the prefectures of Japan.

The NHNS database of Japan was used (open data URL; https://www.nibiohn.go.jp/eiken/kenkounippon21/en/eiyouchousa/index.html). Ikeda previously described the sampling design of the Japan NHNS as well as the survey frequency and response rate [2]. The 2012 and 2016 surveys involved a considerable number of samples, including data for participants aged 1 year or older with diabetes and regional disparities in physical characteristics and lifestyle habits across 47 prefectures. Therefore, for 2012 and 2016, data disaggregated by prefecture were compiled to observe exercise habits and step counts. However, owing to the impact of the 2016 earthquake, the survey was not conducted in Kumamoto Prefecture. Furthermore, we explored historical progress in relevant laws and regulations related to the survey results as well as their practical implications. A map of the 47 prefectures of Japan (Fig. 1) was visualized using the ‘ggplot2’ package of R, and the ‘mapdata’ and ‘maps’ packages were used to capture and process data, as described by Lemenkova and Debeir [6]. The scripts are listed in the Supplementary.

### 3.1 Overview

The NHNS had a cross-sectional design and was carried out in regional health centers designated by the Ministry of Health, Labour and Welfare (MHLW). The NHNS comprises three component surveys: physical, nutritional, dietary, and lifestyle habits. Interviewers were mainly dietitians and registered dietitians for the dietary intake survey. Medical doctors, public health nurses, and clinical laboratory technologists performed the physical examinations.

### 3.2 Exercise habits

Since 1986, the presence or absence of exercise habits has been investigated as one item related to physical condition. Regarding the presence or absence of exercise habits, individuals who met all of the following three criteria were defined as having exercise habits: “exercise frequency of ≥2 times a week,” “duration of ≥30 min at a one occasion,” and “continuation of ≥1 year.” This variable was assessed through face-to-face structured interviews, and participants were asked to provide “yes” or “no” responses. From 1995 to 2012, the survey method was modified slightly as follows. Participants were asked to select one item from the three options that best described their exercise habits: 1) inability to exercise due to a health problem, 2) inability to exercise due to other problems, and 3) exercised for at least 30 min per day, at least 2 days per week, over the previous year. Since 2013, participants reported their exercise frequency (days/week), duration (minutes/day), and period (years), provided that it was not contraindicated by their physician. From 2013 onwards, those who answered “yes” to “currently prohibited from exercising by doctors” did not need to respond to “exercise habits.” From the three questions, “Number of days of exercise per week,” “Average duration of exercise per day,” and “Number of years of continuous exercise,” “≥2 days per week as frequency of exercise,” “Average duration of exercise per day of 30 min,” and “>1 year of continuous exercise” were defined as habitual exercisers. Questions regarding exercise habits were not asked using validated questions, and questions about exercise habits differed slightly before and after 2013. However, an exercise habit itself has been consistently defined since the start of the survey as “individuals who exercise for ≥30 min at least twice a week for at least a year.” The crude and age-adjusted values are published on the website.

### 3.3 Step counts

Following exercise habits determined using a questionnaire, pedometer-based surveillance was first implemented in 1989 to collect objective information on physical activity among adults aged ≥30 years. The pedometer (ALNESS 200 S AS-200, Yamasa Co., Tokyo, Japan), worn at the waist, was used to measure daily step counts for November, excluding Sundays and holidays. All participants recorded their own step counts using a diary because a Yamasa pedometer has no memory function. Regarding the aggregated data, individuals with fewer than 100 steps or over 50,000 steps were excluded.

### 3.4 Sedentary behavior

In the NHNS, questionnaire-based surveys on sitting time and sedentary activity were conducted three times in 2006, 2013, and 2017. The methods used in the three surveys were as follows. In the 2006 and 2013 surveys, participants were asked, “On average, how much time do you spend sitting or lying down during the day?” The sitting or lying down time included the time spent sitting at a desk or computer (including work, study, and reading), watching TV, sitting and conversing, driving a car (or riding in a car), and sitting on a train. However, sleep duration was not included. Participants provided numerical values for the times on weekdays and weekends. This questionnaire is the same as the IPAQ Short Form [7]. Data are presented in seven categories (0 min, 1 min to <2 h, 2 to <4 h, 4 to <6 h, 6 to <8 h, 8 to <10 h, and ≥10 h). In a 2017 survey, participants were asked, “How long on average do you engage in the following activities each day: 1) physical labor or sports, 2) sitting, 3) standing or walking?” For the sitting category, participants were given three options: 1) <3 h, 2) to 3–8 h, and 3) ≥8 h. These questions are the same as those in the 5-year follow-up survey of the Japan Public Health Center-based Prospective Study [8].

## 4 Results

### 4.1 Exercise habit

**Fig. 2:**
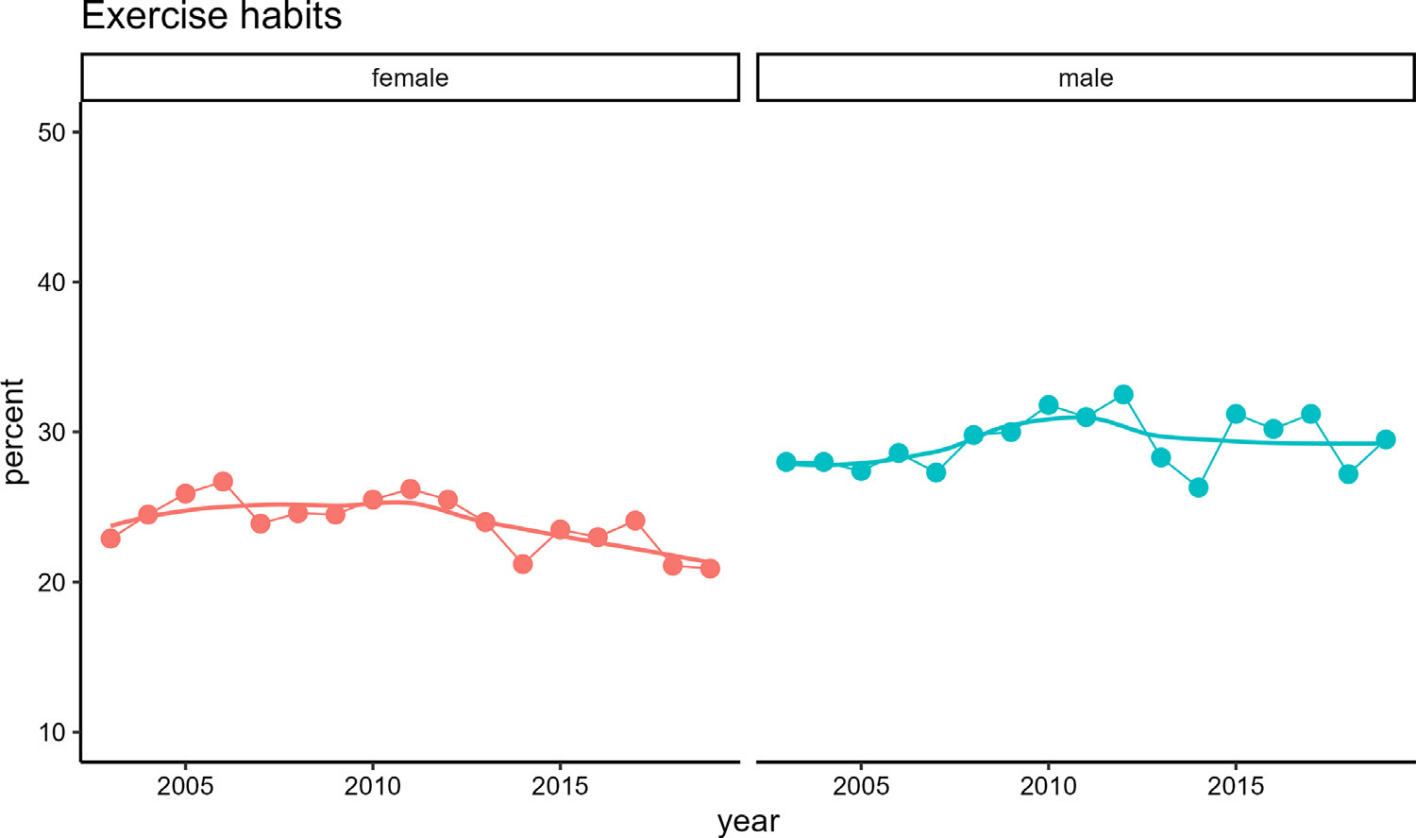
Trends in the age-adjusted prevalence of exercise habits by gender from 2003 to 2019, based on the [2003–2019_exercise.csv] dataset.

Fig. 2 shows the trends in the age-adjusted prevalence of exercise habits by gender from 2003 to 2019. According to the 2019 NHNS conducted by the Ministry of Health, Labour, and Welfare in the first year of the Reiwa era, the prevalence of exercise habits was 20.9% for female and 29.5% for male. Over the past two decades, there was no significant increase or decrease in prevalence for male and a significant decrease in that for female (22.9%).

**Fig. 3:**
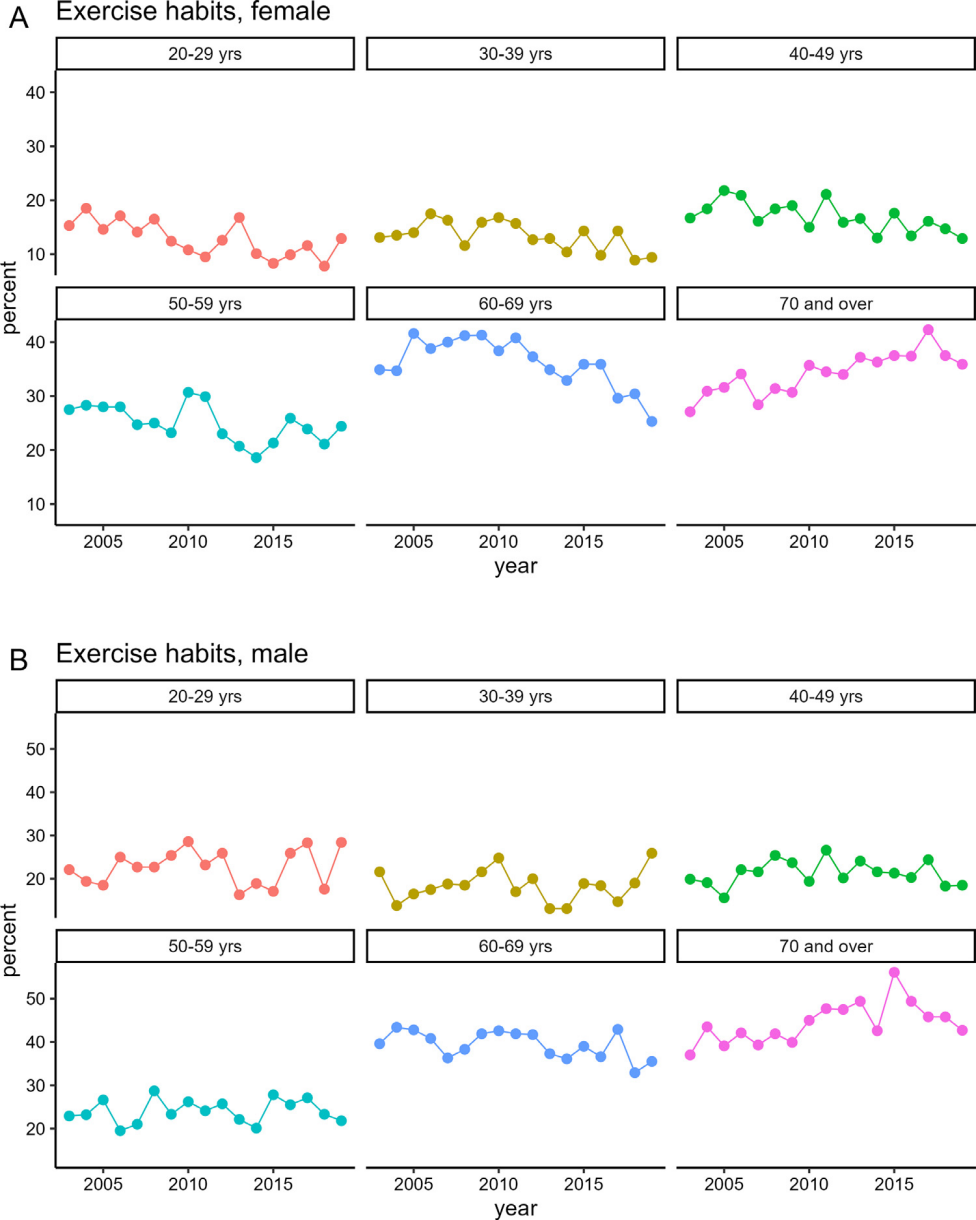
Trends in the prevalence of exercise habits according to gender and age groups from 2003 to 2019, based on the [2003–2019_exercise.csv] dataset.

The prevalence of exercise habits according to gender and age groups is shown in Fig. 3a and b, respectively. When viewed by age group, both female and male exhibited a prevalence of exercise habits of <30% in younger and middle-aged generations (20–59 years), whereas individuals aged ≥60 years showed a higher prevalence than younger and middle-aged generations (20–59 years). Male and female aged ≥70 years exhibited an increasing trend since 2003. However, in the age group of 60–69 years, female experienced a significant decline. For the third term of Health Japan 21, scheduled to start in 2024, the target percentages of individuals with regular exercise habits are 30% for those aged 20–64 years and 50% for those aged ≥65 years.

### 4.2 Step counts

**Fig. 4:**
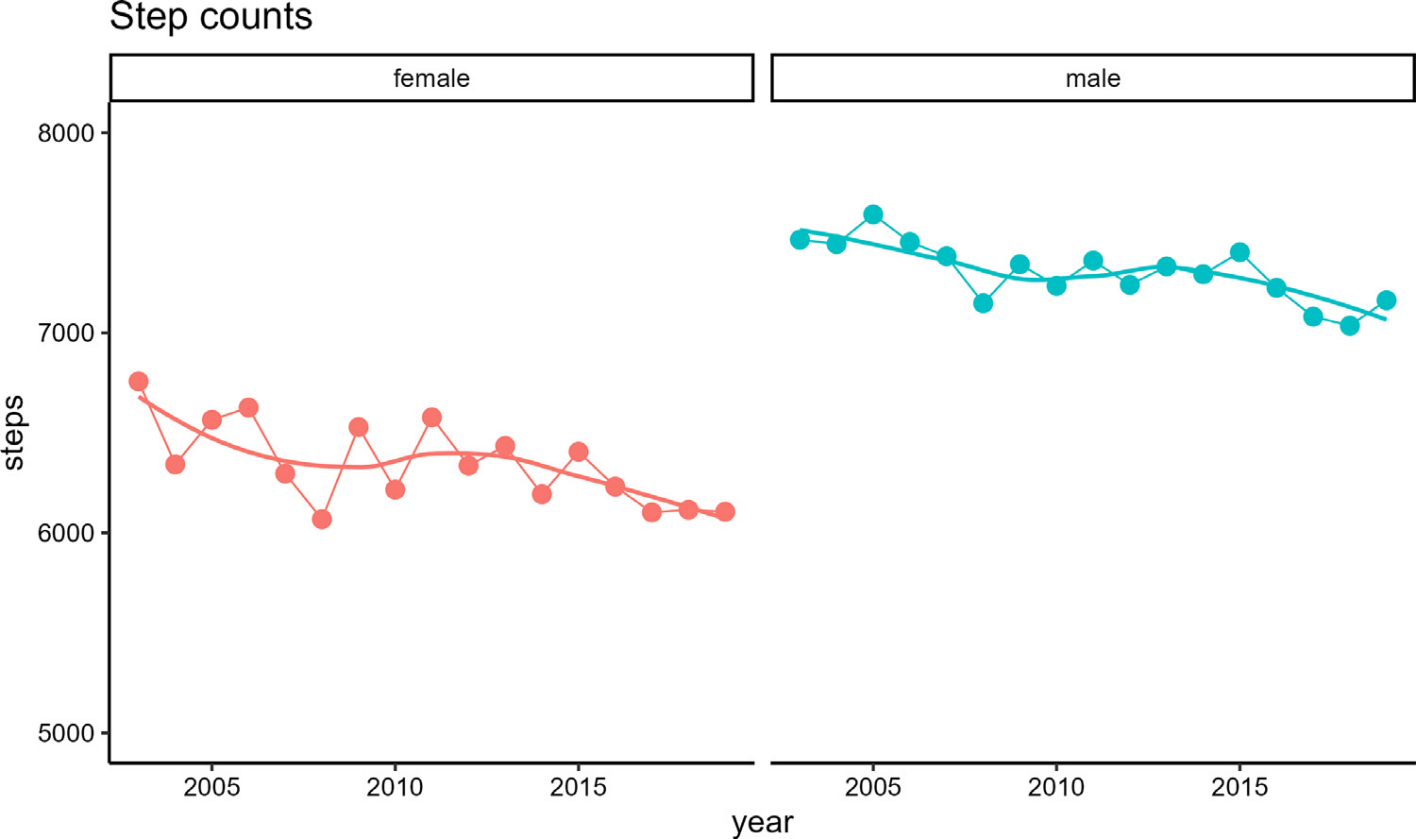
Trends in the age-adjusted pedometer-measured step counts by gender from 2003 to 2019, based on the [1989–2019_step.csv] dataset.

Fig. 4 presents age-adjusted pedometer-measured step counts by gender from 2003 to 2019 for those aged >20 years. The average numbers of steps taken were 7162 for male and 6105 for female in the 2019 survey. Over the past decade, there has been a gradual decrease in step count for male, from 7465 in 2003, while female experienced a significant decline from 6757 in 2003.

**Fig. 5:**
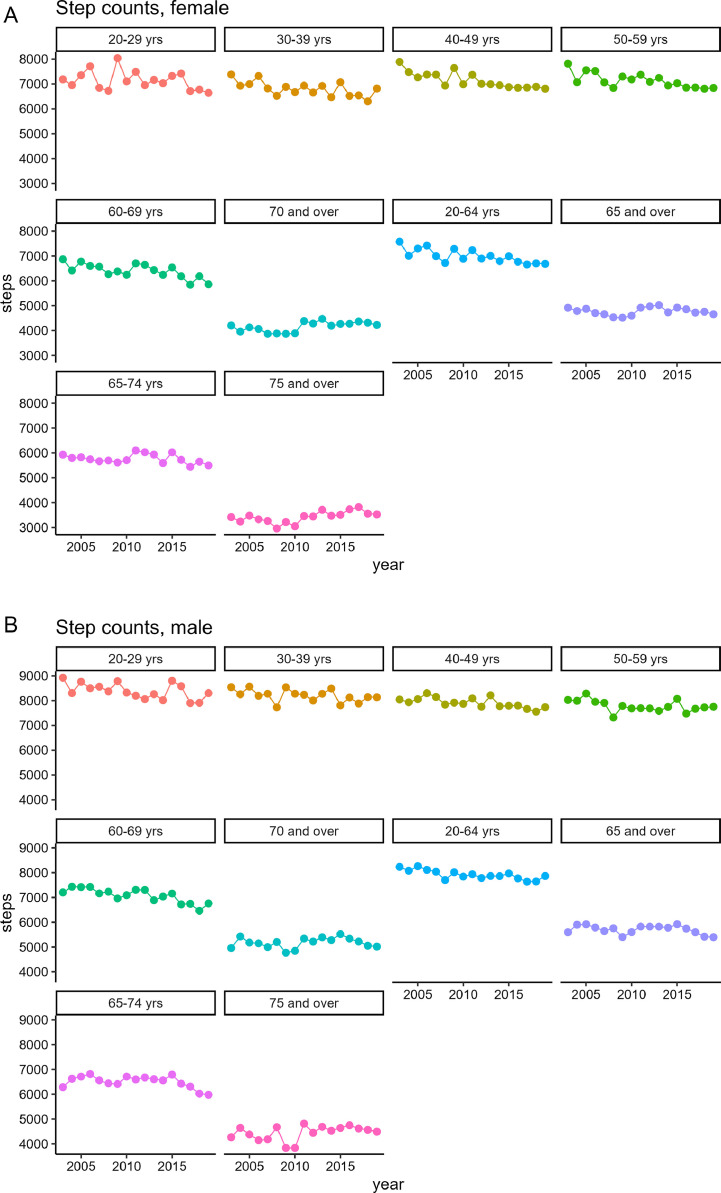
Trends in the pedometer-measured step counts according to gender and age groups from 2003 to 2019, based on the [1989–2019_step.csv] dataset.

There has been a decrease or stagnation in step counts for both age groups and a decrease in steps for female aged 20–64 years in an analysis of long-term trends (Fig. 5a and b). For the third term of Health Japan 21, the target step counts are 8000 steps for those aged 20–64 years and 6000 steps for those aged ≥65 years.

**Fig. 6:**
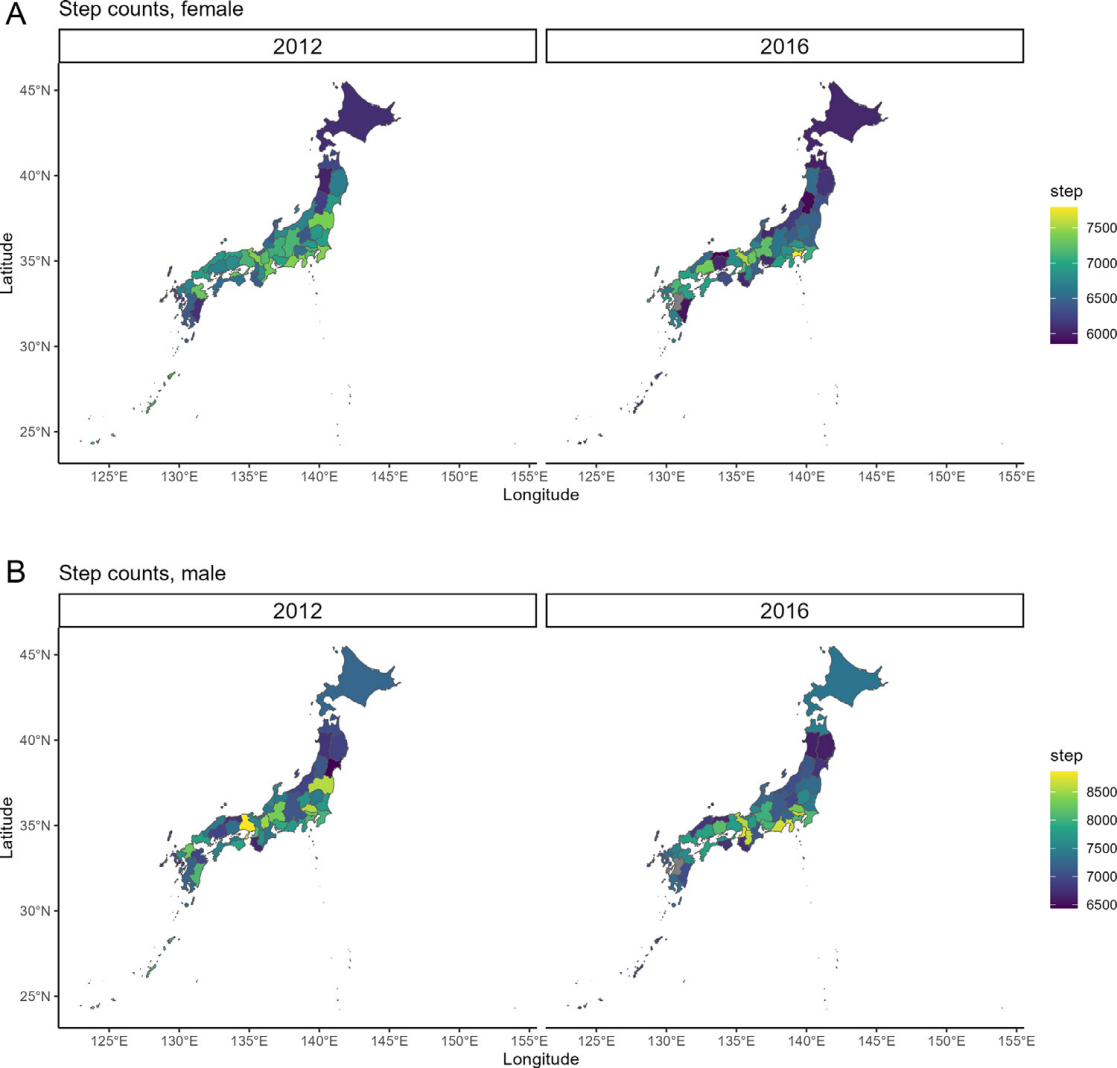
The pedometer-measured step counts according to gender and prefecture in 2012 and 2016, based on the [2012_step_pref.csv and 2016_step_pref.csv] dataset. Owing to the impact of the 2016 earthquake, the survey was not conducted in Kumamoto Prefecture.

Fig. 6a and b displays maps illustrating the prefecture-specific step counts by gender for 2012 and 2016. In 2016, data for Kumamoto Prefecture were not available. Major metropolitan areas, such as the Greater Tokyo Area, Aichi, and Osaka, generally exhibited higher step counts than those in other regions. Ihara et al. [9] examined the association between city scale and daily steps from data for 15,763 male and 18,479 female aged ≥20 years who participated in a 1-day pedometer measurement at any point during the NHNS between 2006 and 2010. They reported that individuals residing in larger cities, irrespective of gender, had higher step counts than individuals living in smaller cities. Additionally, regardless of age or employment status, subgroup analyses revealed significant differences in mean daily step counts between urban and non-urban settings for both genders.

### 4.3 Sedentary behavior

#### 4.3.1 Survey in 2006

**Fig. 7:**
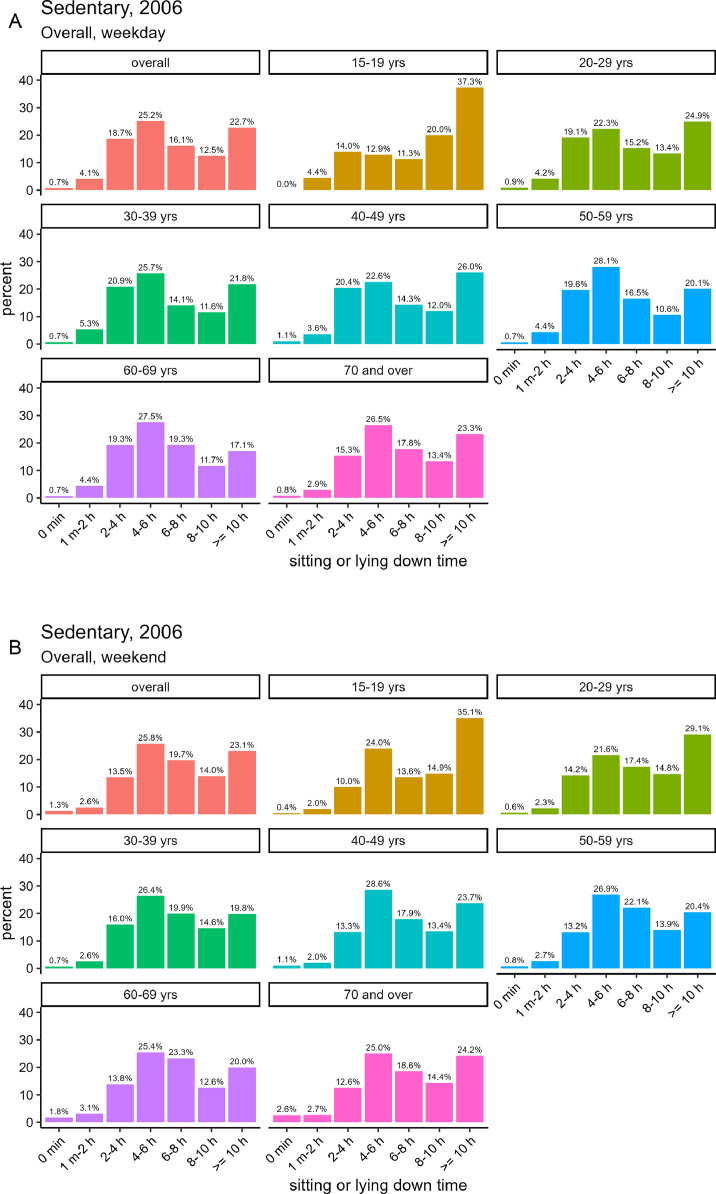
The total hours of sitting or lying down time per day by gender in the NHNS in 2006, based on the [2006_sedentary.csv] dataset.

Fig. 7a and b shows the total hours of sitting or lying down time per day in the NHNS in 2006 (with valid responses from 8190 participants, including 3786 male and 4404 female). The results for the >8 h group indicate that on weekdays, sitting time was 35.2% overall, 37.3% for male, and 33.4% for female. On the weekends, the corresponding values were 37.1% overall, 40.9% for male, and 33.8% for female.

#### 4.3.2 Survey in 2013

**Fig. 8:**
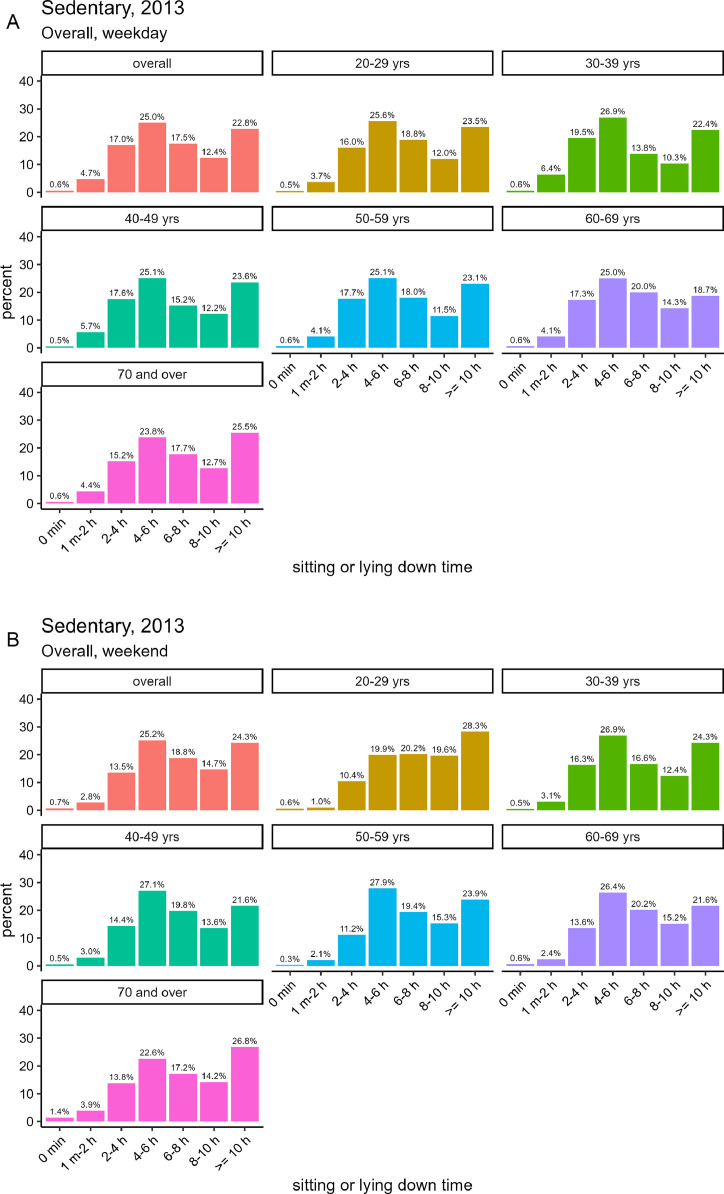
The total hours of sitting or lying down time per day by gender in the NHNS in 2013, based on the [2013_sedentary.csv] dataset.

Fig. 8a and b illustrates the daily total sitting or lying down time in the 2013 NHNS, segmented by weekdays and weekends (based on valid responses from 7082 participants, including 3290 male and 3792 female). On weekdays, the percentage of respondents reporting sitting for ≥8 h was 35.2% overall, with male exhibiting a higher percentage (38.1%) than female (32.8%). In contrast, the percentage of individuals who reported sitting for ≥8 h during the weekend was 39.0% overall (42.8% for male and 35.7% for female). Thus, both male and female tended to have longer total hours of sitting on weekends than on weekdays.

#### 4.3.3 Survey in 2017

**Fig. 9:**
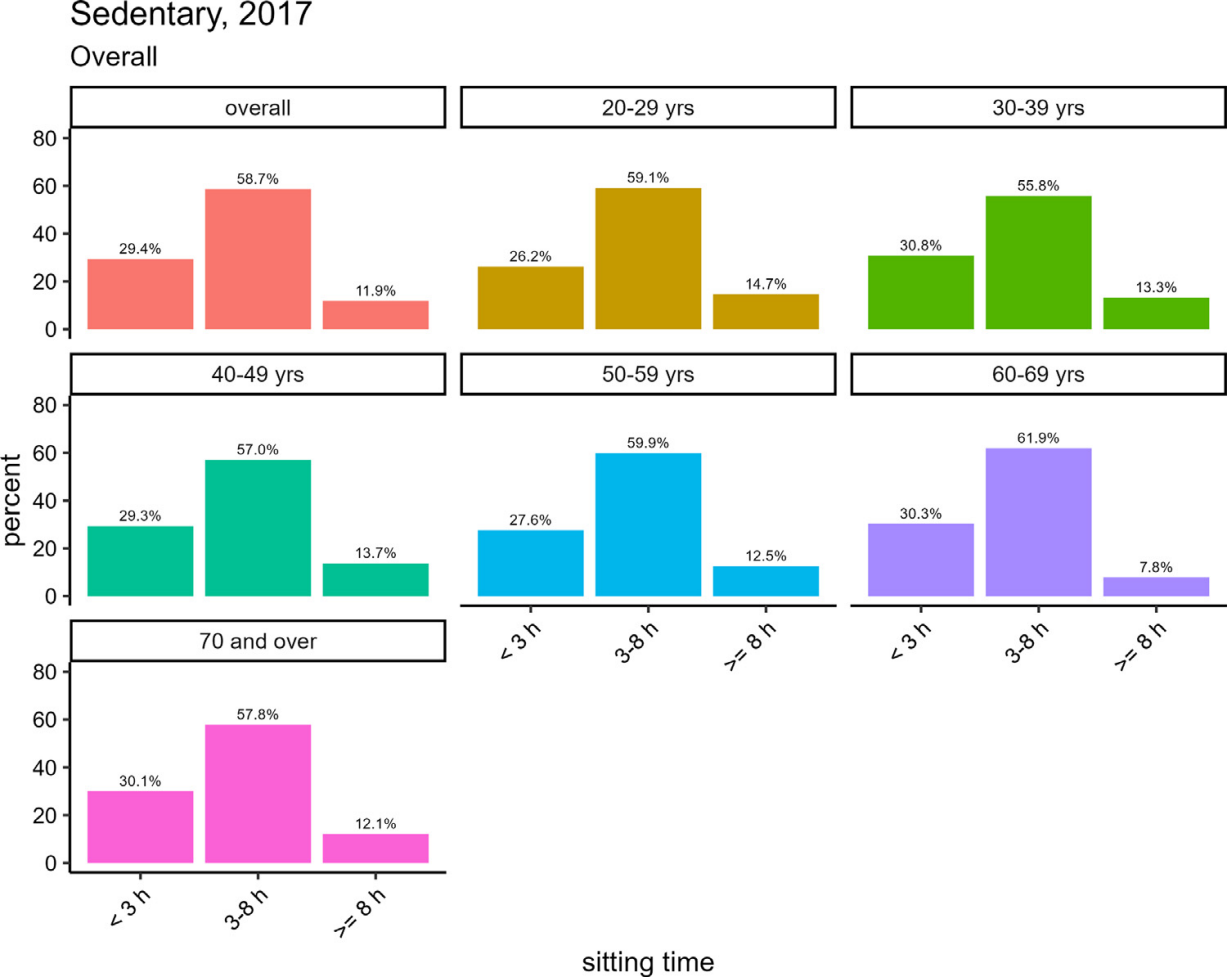
The total hours of sitting or lying down time per day by gender in the NHNS in 2017, based on the [2017_sedentary.csv] dataset.

Fig. 9 shows the total hours of sitting time per day in the NHNS of 2017 (with valid responses from 6565 participants, including 3096 male and 3468 female), the proportion of participants spending ≥8 h h per day sitting was 11.9% overall (13.8% for male and 10.3% for female). When considering gender differences, for male, the age groups of 60–69 years and ≥70 years showed lower proportions of sitting for ≥8 h (10.3% and 12.3%, respectively) than the age group of 20–59 years (15.0–17.4%). Among female, the proportion in the 60-year-old group was the lowest at 5.5%, whereas that for other age groups was 10–12%.

Supplementary Table 1–3 shows the data of sitting time and sedentary behavior in 2006, 2013, and 2017 according to gender and age categories.

## Limitations

First, we were unable to obtain additional data on the participants of this cross-sectional survey, as the data was collected from electronically available aggregated reports on the official website of the Ministry of Health, Labour, and Welfare of Japan. Second, the low response rates, approximately 50–55%, may impact the representativeness of the sample and the reliability of population estimates. Notably, the response rate of males was lower than that of females at the individual level, and the response rate of both gender in the older age groups was higher than that of those in their 20 s. This suggests that they may have been more health-conscious than the general population, indicating the possibility of a selection bias.

## Ethics Statement

This study followed the ethical requirements for publication in Data in Brief. Based on the Health Promotion Act (Act No. 103 of 2002), the National Health and Nutrition Survey is a survey conducted annually by the Ministry of Health, Labour, and Welfare to understand the status of public health, nutritional intake, and lifestyle habits and obtain basic data necessary for comprehensive health promotion. Data were collected from electronically available aggregated reports from the official website of the Ministry of Health, Labour, and Welfare of Japan. The National Health and Nutrition Survey, Japan, was conducted according to the guidelines laid down in the Declaration of Helsinki. In the National Health and Nutrition Survey, Japan, study procedures and the risks associated with participating were explained, and written informed consent was obtained from all participants. An ethics review from the National Institute of Health and Nutrition was deemed unnecessary because we used only data of annual reports and summary tables that were anonymized and contained no information that could be used to identify the individuals.

## CRediT authorship contribution statement

**Takashi Nakagata:** Conceptualization, Methodology, Data curation, Visualization, Writing – original draft, Writing – review & editing. **Rei Ono:** Writing – review & editing, Funding acquisition.

## Data Availability

National Health and Nutrition Survey (Reference data).

## Appendix Supplementary materials

Image, application 1
