# Supplementary material for: Data resource profile: Exercise habits, step counts, and sedentary behavior from the National Health and Nutrition Survey in Japan
Source: Data Brief. 2024 Jan 24;53:110103. doi: 10.1016/j.dib.2024.110103 (PMC10847803; doi:10.1016/j.dib.2024.110103)
Supplement: Supplementary file 1 [file mmc1.docx]

Supplementary

R script for plotting prefectures of Japan.

# japan map ----------------------------------------------------------------------------

japan <- map_data ("japan")

nb_cols <- 47 # 61: Corrected variable name

mycolors <- colorRampPalette(brewer.pal(8, "Set1"))(nb_cols)

print(japan)

str(japan)

colnames(japan)

# Create a ggplot with 47 colors

# Use scale_fill_manual

japan.map <- ggplot() +

geom_polygon(data = japan, aes(x = long, y = lat, fill = region, group = group),

color = "black", linetype = 1, size = 0.2) +

coord_fixed(1.3, xlim = c(120, 150)) +

xlab("Longitude") + # 69: Corrected label

ylab("Latitude") + # 70: Corrected label

scale_fill_manual(values = mycolors) + # 71: Corrected function name

labs(title = "Japan",colour = "black",size = 16,

subtitle = "Mapping: R", colour = "black",

caption = "Packages: ggmap, ggplot2, mapdata, maps", colour = "black") +

theme(legend.title = element_text(colour = "black", size = 16, face = "bold"),

legend.text = element_text(colour = "black",size = 14),

plot.title = element_text(face = "bold", colour = "black", size = 16), # 75: Corrected family

axis.text.x = element_text(color = "black", size = 14),

axis.text.y = element_text(color = "black", size = 14),

axis.title.x = element_text(size = 16),

axis.title.y = element_text(size = 16),

# plot.subtitle, plot.caption, and other theme settings...

) +

guides(fill = guide_legend(ncol = 2, title = "Prefectures", title.position = "top"))

print(japan.map)

# finish ----------------------------------------------------------------------------

Supplementary Table 1. Total hours of sitting or lying down time per day in the NHNS in 2006

| Day | Gender | Age group | Time | | | | | | |
| --- | --- | --- | --- | --- | --- | --- | --- | --- | --- |
|  |  |  | 0 min | 1 m–2 h | 2–4 h | 4–6 h | 6–8 h | 8–10 h | ≥10 h |
| weekday | overall | overall | 0.7 (60) | 4.1 (336) | 18.7 (1528) | 25.2 (2060) | 16.1 (1322) | 12.5 (1021) | 22.7 (1863) |
|  |  | 15–19 yrs | 0.0 (0) | 4.4 (20) | 14.0 (63) | 12.9 (58) | 11.3 (51) | 20.0 (90) | 37.3 (168) |
|  |  | 20–29 yrs | 0.9 (7) | 4.2 (33) | 19.1 (152) | 22.3 (177) | 15.2 (121) | 13.4 (106) | 24.9 (198) |
|  |  | 30–39 yrs | 0.7 (9) | 5.3 (68) | 20.9 (267) | 25.7 (329) | 14.1 (180) | 11.6 (148) | 21.8 (279) |
|  |  | 40–49 yrs | 1.1 (12) | 3.6 (41) | 20.4 (233) | 22.6 (258) | 14.3 (163) | 12.0 (137) | 26.0 (297) |
|  |  | 50–59 yrs | 0.7 (10) | 4.4 (66) | 19.6 (297) | 28.1 (425) | 16.5 (250) | 10.6 (160) | 20.1 (304) |
|  |  | 60–69 yrs | 0.7 (9) | 4.4 (60) | 19.3 (262) | 27.5 (374) | 19.3 (262) | 11.7 (159) | 17.1 (232) |
|  |  | 70 and over | 0.8 (13) | 2.9 (48) | 15.3 (254) | 26.5 (439) | 17.8 (295) | 13.4 (221) | 23.3 (385) |
|  | female | overall | 0.6 (26) | 3.8 (169) | 18.5 (814) | 26.4 (1164) | 17.2 (758) | 13.1 (579) | 20.3 (894) |
|  |  | 15–19 yrs | 0.0 (0) | 3.7 (8) | 9.3 (20) | 11.1 (24) | 9.3 (20) | 21.8 (47) | 44.9 (97) |
|  |  | 20–29 yrs | 0.5 (2) | 3.3 (14) | 16.6 (71) | 21.0 (90) | 17.2 (74) | 16.6 (71) | 24.9 (107) |
|  |  | 30–39 yrs | 0.4 (3) | 4.8 (33) | 21.9 (151) | 28.4 (196) | 14.1 (97) | 12.2 (84) | 18.3 (126) |
|  |  | 40–49 yrs | 1.0 (6) | 3.8 (23) | 22.3 (135) | 24.5 (148) | 14.7 (89) | 12.6 (76) | 21.2 (128) |
|  |  | 50–59 yrs | 1.1 (8) | 5.3 (38) | 19.2 (137) | 25.8 (184) | 13.5 (96) | 11.5 (82) | 23.6 (168) |
|  |  | 60–69 yrs | 0.5 (4) | 4.6 (34) | 19.5 (144) | 29.0 (214) | 20.5 (151) | 12.8 (94) | 13.0 (96) |
|  |  | 70 and over | 1.0 (9) | 3.1 (29) | 14.3 (133) | 27.0 (251) | 18.6 (173) | 13.9 (129) | 22.0 (204) |
|  | male | overall | 0.9 (34) | 4.4 (167) | 18.9 (714) | 23.7 (896) | 14.9 (564) | 11.7 (442) | 25.6 (969) |
|  |  | 15–19 yrs | 0.0 (0) | 5.1 (12) | 18.4 (43) | 14.5 (34) | 13.2 (31) | 18.4 (43) | 30.3 (71) |
|  |  | 20–29 yrs | 1.4 (5) | 5.2 (19) | 22.2 (81) | 23.8 (87) | 12.9 (47) | 9.6 (35) | 24.9 (91) |
|  |  | 30–39 yrs | 1.0 (6) | 5.9 (35) | 19.7 (116) | 22.5 (133) | 14.1 (83) | 10.8 (64) | 25.9 (153) |
|  |  | 40–49 yrs | 1.1 (6) | 3.4 (18) | 18.3 (98) | 20.5 (110) | 13.8 (74) | 11.4 (61) | 31.5 (169) |
|  |  | 50–59 yrs | 1.1 (8) | 5.3 (38) | 19.2 (137) | 25.8 (184) | 13.5 (96) | 11.5 (82) | 23.6 (168) |
|  |  | 60–69 yrs | 0.8 (5) | 4.2 (26) | 19.0 (118) | 25.8 (160) | 17.9 (111) | 10.5 (65) | 21.9 (136) |
|  |  | 70 and over | 0.6 (4) | 2.6 (19) | 16.6 (121) | 25.9 (188) | 16.8 (122) | 12.7 (92) | 24.9 (181) |
| weekend | overall | overall | 1.3 (107) | 2.6 (209) | 13.5 (1107) | 25.8 (2104) | 19.7 (1613) | 14.0 (1141) | 23.1 (1889) |
|  |  | 15–19 yrs | 0.4 (2) | 2.0 (9) | 10.0 (45) | 24.0 (108) | 13.6 (61) | 14.9 (67) | 35.1 (158) |
|  |  | 20–29 yrs | 0.6 (5) | 2.3 (18) | 14.2 (113) | 21.6 (171) | 17.4 (138) | 14.8 (117) | 29.1 (231) |
|  |  | 30–39 yrs | 0.7 (9) | 2.6 (33) | 16.0 (205) | 26.4 (338) | 19.9 (255) | 14.6 (187) | 19.8 (253) |
|  |  | 40–49 yrs | 1.1 (12) | 2.0 (23) | 13.3 (151) | 28.6 (325) | 17.9 (204) | 13.4 (153) | 23.7 (270) |
|  |  | 50–59 yrs | 0.8 (12) | 2.7 (40) | 13.2 (199) | 26.9 (405) | 22.1 (334) | 13.9 (210) | 20.4 (308) |
|  |  | 60–69 yrs | 1.8 (24) | 3.1 (42) | 13.8 (187) | 25.4 (344) | 23.3 (315) | 12.6 (170) | 20.0 (270) |
|  |  | 70 and over | 2.6 (43) | 2.7 (44) | 12.6 (207) | 25.0 (413) | 18.6 (306) | 14.4 (237) | 24.2 (399) |
|  | female | overall | 1.4 (62) | 3.1 (135) | 14.0 (616) | 27.0 (1185) | 20.7 (911) | 13.6 (599) | 20.2 (887) |
|  |  | 15–19 yrs | 0.9 (2) | 1.4 (3) | 9.3 (20) | 23.1 (50) | 14.4 (31) | 11.6 (25) | 39.4 (85) |
|  |  | 20–29 yrs | 0.7 (3) | 2.3 (10) | 12.4 (53) | 20.1 (86) | 18.9 (81) | 15.4 (66) | 30.1 (129) |
|  |  | 30–39 yrs | 0.4 (3) | 3.8 (26) | 17.5 (121) | 29.3 (202) | 20.6 (142) | 13.3 (92) | 15.1 (104) |
|  |  | 40–49 yrs | 1.3 (8) | 2.6 (16) | 14.0 (85) | 32.4 (196) | 18.2 (110) | 12.7 (77) | 18.7 (113) |
|  |  | 50–59 yrs | 0.6 (5) | 2.5 (20) | 14.4 (115) | 29.2 (233) | 24.0 (191) | 12.8 (102) | 16.4 (131) |
|  |  | 60–69 yrs | 2.2 (16) | 4.0 (29) | 14.9 (109) | 26.3 (193) | 24.4 (179) | 13.0 (95) | 15.3 (112) |
|  |  | 70 and over | 2.7 (25) | 3.3 (31) | 12.2 (113) | 24.3 (225) | 19.1 (177) | 15.3 (142) | 23.0 (213) |
|  | male | overall | 1.2 (45) | 2.0 (74) | 13.0 (491) | 24.3 (919) | 18.6 (702) | 14.4 (542) | 26.5 (1002) |
|  |  | 15–19 yrs | 0.0 (0) | 2.6 (6) | 10.7 (25) | 24.8 (58) | 12.8 (30) | 17.9 (42) | 31.2 (73) |
|  |  | 20–29 yrs | 0.5 (2) | 2.2 (8) | 16.4 (60) | 23.3 (85) | 15.6 (57) | 14.0 (51) | 27.9 (102) |
|  |  | 30–39 yrs | 1.0 (6) | 1.2 (7) | 14.2 (84) | 23.1 (136) | 19.2 (113) | 16.1 (95) | 25.3 (149) |
|  |  | 40–49 yrs | 0.8 (4) | 1.3 (7) | 12.4 (66) | 24.2 (129) | 17.6 (94) | 14.3 (76) | 29.5 (157) |
|  |  | 50–59 yrs | 1.0 (7) | 2.8 (20) | 11.8 (84) | 24.2 (172) | 20.1 (143) | 15.2 (108) | 24.9 (177) |
|  |  | 60–69 yrs | 1.3 (8) | 2.1 (13) | 12.6 (78) | 24.4 (151) | 22.0 (136) | 12.1 (75) | 25.5 (158) |
|  |  | 70 and over | 2.5 (18) | 1.8 (13) | 13.0 (94) | 26.0 (188) | 17.8 (129) | 13.1 (95) | 25.7 (186) |

Data are expressed as percent (number).

Supplementary Table 2. Total hours of sitting or lying down time per day in the NHNS in 2013

| Day | Gender | Age group | Time | | | | | | |
| --- | --- | --- | --- | --- | --- | --- | --- | --- | --- |
|  |  |  | 0 min | 1 m–2 h | 2–4 h | 4–6 h | 6–8 h | 8–10 h | ≥10 h |
| weekday | overall | overall | 0.6 (41) | 4.7 (331) | 17.0 (1203) | 25 (1771) | 17.5 (1237) | 12.4 (881) | 22.8 (1618) |
|  |  | 20–29 yrs | 0.5 (3) | 3.7 (23) | 16.0 (99) | 25.6 (158) | 18.8 (116) | 12.0 (74) | 23.5 (145) |
|  |  | 30–39 yrs | 0.6 (5) | 6.4 (56) | 19.5 (170) | 26.9 (234) | 13.8 (120) | 10.3 (90) | 22.4 (195) |
|  |  | 40–49 yrs | 0.5 (6) | 5.7 (63) | 17.6 (193) | 25.1 (276) | 15.2 (167) | 12.2 (134) | 23.6 (259) |
|  |  | 50–59 yrs | 0.6 (6) | 4.1 (43) | 17.7 (186) | 25.1 (264) | 18.0 (189) | 11.5 (121) | 23.1 (243) |
|  |  | 60–69 yrs | 0.6 (9) | 4.1 (62) | 17.3 (263) | 25.0 (381) | 20.0 (304) | 14.3 (218) | 18.7 (285) |
|  |  | 70 and over | 0.6 (12) | 4.4 (84) | 15.2 (292) | 23.8 (458) | 17.7 (341) | 12.7 (244) | 25.5 (491) |
|  | female | overall | 0.4 (16) | 4.7 (177) | 17.1 (650) | 26.5 (1004) | 18.5 (701) | 12.5 (475) | 20.3 (769) |
|  |  | 20–29 yrs | 0.3 (1) | 2.5 (8) | 17.4 (55) | 22.1 (70) | 19.9 (63) | 12.3 (39) | 25.6 (81) |
|  |  | 30–39 yrs | 0.0 (0) | 6.6 (30) | 19.1 (87) | 30.0 (137) | 14.5 (66) | 10.7 (49) | 19.1 (87) |
|  |  | 40–49 yrs | 0.3 (2) | 5.6 (33) | 16.9 (99) | 27.9 (164) | 16.5 (97) | 12.4 (73) | 20.3 (119) |
|  |  | 50–59 yrs | 0.4 (2) | 4.1 (23) | 18.5 (103) | 28.2 (157) | 18.3 (102) | 11.7 (65) | 18.7 (104) |
|  |  | 60–69 yrs | 0.5 (4) | 3.9 (32) | 17.4 (144) | 27.7 (229) | 21.9 (181) | 14.3 (118) | 14.4 (119) |
|  |  | 70 and over | 0.7 (7) | 4.9 (51) | 15.4 (162) | 23.5 (247) | 18.3 (192) | 12.5 (131) | 24.7 (259) |
|  | male | overall | 0.8 (25) | 4.7 (154) | 16.8 (553) | 23.3 (767) | 16.3 (536) | 12.3 (406) | 25.8 (849) |
|  |  | 20–29 yrs | 0.7 (2) | 5.0 (15) | 14.6 (44) | 29.2 (88) | 17.6 (53) | 11.6 (35) | 21.3 (64) |
|  |  | 30–39 yrs | 1.2 (5) | 6.3 (26) | 20.0 (83) | 23.4 (97) | 13.0 (54) | 9.9 (41) | 26.1 (108) |
|  |  | 40–49 yrs | 0.8 (4) | 5.9 (30) | 18.4 (94) | 21.9 (112) | 13.7 (70) | 11.9 (61) | 27.4 (140) |
|  |  | 50–59 yrs | 0.8 (4) | 4.0 (20) | 16.7 (83) | 21.6 (107) | 17.5 (87) | 11.3 (56) | 28.0 (139) |
|  |  | 60–69 yrs | 0.7 (5) | 4.3 (30) | 17.1 (119) | 21.9 (152) | 17.7 (123) | 14.4 (100) | 23.9 (166) |
|  |  | 70 and over | 0.6 (5) | 3.8 (33) | 14.9 (130) | 24.2 (211) | 17.1 (149) | 12.9 (113) | 26.6 (232) |
| weekend | overall | overall | 0.7 (52) | 2.8 (200) | 13.5 (955) | 25.2 (1785) | 18.8 (1329) | 14.7 (1043) | 24.3 (1718) |
|  |  | 20–29 yrs | 0.6 (4) | 1.0 (6) | 10.4 (64) | 19.9 (123) | 20.2 (125) | 19.6 (121) | 28.3 (175) |
|  |  | 30–39 yrs | 0.5 (4) | 3.1 (27) | 16.3 (142) | 26.9 (234) | 16.6 (144) | 12.4 (108) | 24.3 (211) |
|  |  | 40–49 yrs | 0.5 (6) | 3.0 (33) | 14.4 (158) | 27.1 (298) | 19.8 (217) | 13.6 (149) | 21.6 (237) |
|  |  | 50–59 yrs | 0.3 (3) | 2.1 (22) | 11.2 (118) | 27.9 (293) | 19.4 (204) | 15.3 (161) | 23.9 (251) |
|  |  | 60–69 yrs | 0.6 (9) | 2.4 (37) | 13.6 (207) | 26.4 (402) | 20.2 (308) | 15.2 (231) | 21.6 (328) |
|  |  | 70 and over | 1.4 (26) | 3.9 (75) | 13.8 (266) | 22.6 (435) | 17.2 (331) | 14.2 (273) | 26.8 (516) |
|  | female | overall | 0.8 (29) | 3.4 (130) | 14.5 (551) | 26.3 (997) | 19.3 (733) | 14.3 (542) | 21.4 (810) |
|  |  | 20–29 yrs | 0.3 (1) | 0.9 (3) | 11.7 (37) | 19.9 (63) | 20.8 (66) | 18.3 (58) | 28.1 (89) |
|  |  | 30–39 yrs | 0.0 (0) | 3.9 (18) | 20.2 (92) | 28.7 (131) | 15.6 (71) | 12.7 (58) | 18.9 (86) |
|  |  | 40–49 yrs | 0.5 (3) | 3.9 (23) | 15.7 (92) | 29.6 (174) | 19.1 (112) | 12.8 (75) | 18.4 (108) |
|  |  | 50–59 yrs | 0.2 (1) | 2.5 (14) | 11.5 (64) | 30.0 (167) | 21.4 (119) | 14.2 (79) | 20.1 (112) |
|  |  | 60–69 yrs | 0.7 (6) | 2.9 (24) | 14.8 (122) | 27.2 (225) | 22.0 (182) | 15.6 (129) | 16.8 (139) |
|  |  | 70 and over | 1.7 (18) | 4.6 (48) | 13.7 (144) | 22.6 (237) | 17.4 (183) | 13.6 (143) | 26.3 (276) |
|  | male | overall | 0.7 (23) | 2.1 (70) | 12.3 (404) | 24 (788) | 18.1 (596) | 15.2 (501) | 27.6 (908) |
|  |  | 20–29 yrs | 1.0 (3) | 1.0 (3) | 9.0 (27) | 19.9 (60) | 19.6 (59) | 20.9 (63) | 28.6 (86) |
|  |  | 30–39 yrs | 1.0 (4) | 2.2 (9) | 12.1 (50) | 24.9 (103) | 17.6 (73) | 12.1 (50) | 30.2 (125) |
|  |  | 40–49 yrs | 0.6 (3) | 2.0 (10) | 12.9 (66) | 24.3 (124) | 20.5 (105) | 14.5 (74) | 25.2 (129) |
|  |  | 50–59 yrs | 0.4 (2) | 1.6 (8) | 10.9 (54) | 25.4 (126) | 17.1 (85) | 16.5 (82) | 28.0 (139) |
|  |  | 60–69 yrs | 0.4 (3) | 1.9 (13) | 12.2 (85) | 25.5 (177) | 18.1 (126) | 14.7 (102) | 27.2 (189) |
|  |  | 70 and over | 0.9 (8) | 3.1 (27) | 14 (122) | 22.7 (198) | 17 (148) | 14.9 (130) | 27.5 (240) |

Data are expressed as percent (number).

Supplementary Table 3. Total hours of sitting time per day in the NHNS in 2017

| Gender | Age group | Time | | |
| --- | --- | --- | --- | --- |
|  |  | < 3 h | 3–8 h | ≥8 h |
| overall | overall | 29.4 (1931) | 58.7 (3852) | 11.9 (782) |
|  | 20–29 yrs | 26.2 (130) | 59.1 (293) | 14.7 (73) |
|  | 30–39 yrs | 30.8 (224) | 55.8 (406) | 13.3 (97) |
|  | 40–49 yrs | 29.3 (328) | 57.0 (639) | 13.7 (154) |
|  | 50–59 yrs | 27.6 (271) | 59.9 (589) | 12.5 (123) |
|  | 60–69 yrs | 30.3 (402) | 61.9 (820) | 7.8 (103) |
|  | 70 and over | 30.1 (576) | 57.8 (1105) | 12.1 (232) |
| female | overall | 29.5 (1024) | 60.2 (2089) | 10.3 (356) |
|  | 20–29 yrs | 25.7 (61) | 62.4 (148) | 11.8 (28) |
|  | 30–39 yrs | 33.3 (121) | 55.9 (203) | 10.7 (39) |
|  | 40–49 yrs | 30.3 (177) | 57.9 (338) | 11.8 (69) |
|  | 50–59 yrs | 26.6 (139) | 63.1 (330) | 10.3 (54) |
|  | 60–69 yrs | 30.6 (213) | 63.9 (445) | 5.5 (38) |
|  | 70 and over | 29.4 (313) | 58.6 (625) | 12.0 (128) |
| male | overall | 29.3 (907) | 56.9 (1763) | 13.8 (426) |
|  | 20–29 yrs | 26.6 (69) | 56.0 (145) | 17.4 (45) |
|  | 30–39 yrs | 28.3 (103) | 55.8 (203) | 15.9 (58) |
|  | 40–49 yrs | 28.1 (151) | 56.1 (301) | 15.8 (85) |
|  | 50–59 yrs | 28.7 (132) | 56.3 (259) | 15.0 (69) |
|  | 60–69 yrs | 30.0 (189) | 59.6 (375) | 10.3 (65) |
|  | 70 and over | 31.1 (263) | 56.7 (480) | 12.3 (104) |

Data are expressed as percent (number).
